# Differences in the haematological profile of healthy 70 year old men and women: normal ranges with confirmatory factor analysis

**DOI:** 10.1186/1471-2326-10-4

**Published:** 2010-06-11

**Authors:** Rowan McIlhagger, Alan J Gow, Caroline E Brett, Janie Corley, Michelle Taylor, Ian J Deary, John M Starr

**Affiliations:** 1 Geriatric Medicine unit, University of Edinburgh, Edinburgh, UK; 2 Centre for Cognitive Ageing and Cognitive Epidemiology, University of Edinburgh, Edinburgh, UK; 3 Department of Psychology, University of Edinburgh, Edinburgh, UK

## Abstract

**Background:**

Reference ranges are available for different blood cell counts. These ranges treat each cell type independently and do not consider possible correlations between cell types.

**Methods:**

Participants were identified from the Community Health Index as survivors of the 1947 Scottish Mental Survey, all born in 1936, who were resident in Lothian (potential n = 3,810) and invited to participate in the study. Those who consented were invited to attend a Clinical Research Facility where, amongst other assessments, blood was taken for full blood count. First we described cell count data and bivariate correlations. Next we performed principal components analysis to identify common factors. Finally we performed confirmatory factor analysis to evaluate suitable models explaining relationships between cell counts in men and women.

**Results:**

We examined blood cell counts in 1027 community-resident people with mean age 69.5 (range 67.6-71.3) years. We determined normal ranges for each cell type using Q-Q plots which showed that these ranges were significantly different between men and women for all cell types except basophils. We identified three principal components explaining around 60% of total variance of cell counts. Varimax rotation indicated that these could be considered as erythropoietic, leukopoietic and thrombopoietic factors. We showed that these factors were distinct for men and women by confirmatory factor analysis: in men neutrophil count was part of a 'thrombopoietic' trait whereas for women it was part of a 'leukopoietic' trait.

**Conclusions:**

First, normal ranges for haematological indices should be sex-specific; at present this only pertains to those associated with erythrocytes. Second, differences between individuals across a range of blood cell counts can be explained to a considerable extent by three major components, but these components are not the same in men and women.

## Background

The full blood count is one of the most common investigations patients undergo, and has been available since the 1960s. In adults, reference ranges are the same for all ages despite evidence that erythrocyte count and haemoglobin concentration start to decline in men around 40 years of age; age-associated changes in women are less marked [1]. The World Health Organisation defines anaemia as < 13 g Hb/dL for men and < 12 g Hb/dL for women [2], accepting that women generally have lower haemoglobin concentrations than men. It would be logical to conclude that women of menstruating age are likely to be iron-deficient and therefore have lower haemoglobin concentrations, but studies looking at ferritin levels do not support this [3,4], leading to the suggestion that the difference may be due to hormonal influences on red cell production [5]. In the elderly, there is a significant decline in haemoglobin which, as noted above [1], is more pronounced in men than women [6]. This could be due to falling androgen levels in older men. In previous studies, platelet counts have been found to be significantly higher in women [7,8], with possible explanations of compensation for menstrual blood loss or increased thrombopoietin in women being suggested. One study of healthy Caucasian hospital staff found the total leukocyte count to be significantly higher in women than men due to a highly significant difference in neutrophil count, with no significant correlation between monocytes, basophils and gender [9]. The results of a further study, of staff, students and retired academics, confirmed total leukocyte count to be significantly higher in women than men, but did not examine leukocyte differential to determine the cause of this difference [10]. By contrast, a study of 215 men and 272 women aged 62-90 years found that men had significantly higher total leukocyte counts [11]. Laboratory reference ranges for leukocyte counts often do not differ between men and women. Little is known about whether there are any correlations between the different cell counts in healthy adults. The aims of this study were to, 1) identify any correlations between gender and the components of the full blood count in healthy, community-dwelling elderly people, and 2) examine whether there is a statistically-significant relationship amongthe different cell counts, and whether this relationship is gender-specific.

## Methods

### Sample

Ethics permission for the Lothian Birth Cohort 1936 (LBC1936) study protocol was obtained from the Multi-Centre Research Ethics Committee for Scotland (MREC/01/0/56) and from Lothian Research Ethics Committee (LREC/2003/2/29). The research was carried out in compliance with the Helsinki Declaration. All subjects gave written, informed consent. Participants were recruited from a potential population of 3,810 people identified from the local Community Health Index as born in 1936 and, as such, might have participated in the Scottish Mental Survey 1947. Full details of recruitment and testing have been published previously [12], but of note is a bias to participation by healthier participants who had relatively higher childhood IQ scores. 1,091 participants attended the Wellcome Trust Clinical Research Facility between 2004-2007 for assessment at mean age 69.5 years (minimum 67.6 years, maximum 71.3 years, 90% of participants aged 68.4-70.7 years).

### Measurements

A large number of socio-demographic and health variables were collected at the assessment visit as previously detailed [12]. Blood samples were collected and processed the same day by a LH50 Beckman Coulter instrument. External quality control was provided by UK NEQAS (National External Quality Assessment Service) which includes full blood count each month, and reticulocyte count and differential white cell count every two months. Internal quality control comprised running Coulter 5C single level cell QC and reticulocyte C cell QC first thing each morning. The 5C was repeated at 3.30 pm. Three repeat sample analyses were run at approximately first thing in the morning, lunchtime and early evening. Data were entered into the LBC1936 database and checked [12] with an estimated transcription error rate of 0.13%. Errors were mostly small and not systematic. Finally descriptive statistics were run for all numerical data to ensure that all results were within the appropriate scales, and any errors were checked against the original, handwritten datasheets and corrected as necessary.

### Statistical analysis

All analyses were performed using the SPSS 16.0 and AMOS statistical packages. We used the Kolmogorov-Smirnov statistic to determine whether variables departed from a normal distribution, noting that the value may be more indicative of goodness-of-fit than the p-value in large sample sizes. We used quantile-quantile (Q-Q) plots fitted to a normal distribution to determine 'normal' ranges for each cell count recognising the inadequacy of defining reference ranges in terms of means and standard deviations [1]. We used Varimax rotation in principal components analyses so as to align components most closely with observed variables facilitating more informative confirmatory factor analyses. Principal components analysis is an exploratory procedure that transforms a number of possibly correlated variables into a smaller number of variables called principal components. Principal components analysis makes no assumptions about how the variables might relate to one another: the number of components that emerge is driven by the data. By contrast, confirmatory factor analysis requires that the researcher specifies a model fully describing the hypothesized relationships between variables. This model is formally tested using structural equation modeling (SEM). Goodness-of-fit indices indicate the fit of the pre-specified model to the data set. Another advantage of SEM is that it can be used to test these assumptions in different samples: here we use this approach to test differences in factor structures between men and women. To determine goodness-of-fit for the structural equation models used for confirmatory factor analysis, we used Chi square at p < .05 to accept or reject the null hypothesis that the model provided a poor fit to the data. However, since this might be too conservative for our sample size we also took Root Mean Square Error of Approximation (RMSEA) values < .05 as indicating a good fit, < .08 as indicating an adequate fit and > .1 as indicating a poor fit. We also report the Bentler-Bonnet Index (also known as the Normed Fit Index (NFI)), with values > .95 indicating a good fit and values .90-.95 indicating an adequate fit. For models with more parameters, where the Chi square statistic was significant, we report the Hoelter Index, a measure of sample size adequacy, with values > 200 being acceptable and values < 75 unacceptable.

## Results

### Descriptive data

**Figure 1:**
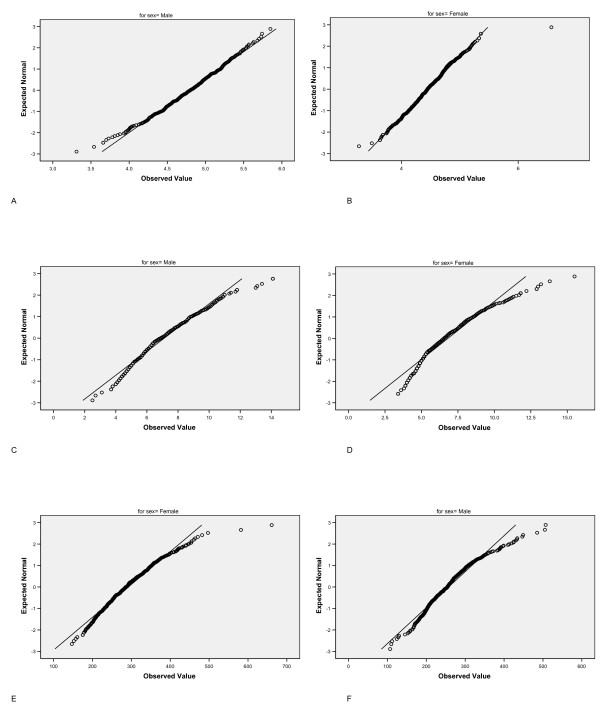
**Q-Q plots for blood cell counts by sex**. Q-Q plots of cell counts against expected normal distribution for 1027 participants aged 70 years for a) erythrocytes (men), b) erythrocytes (women), c) total leukocytes (men), d) total leukocytes (women), e) platelets (men), f) platelets (women).

One thousand and sixty-two (535 male, 527 female) participants, mean age 69.5 (range 67.6-71.3) years, had full blood count data. Participants were mainly from social classes 1-3 with only 4.1% of participants from social classes 4 and 5 (where a higher number denotes a less professional social class). Ten participants were known to have either leukaemia or lymphoma, one participant thrombocytopaenia, and a further 21 participants reported a diagnosis of anaemia and either current or previous iron, vitamin B12 or folic acid treatment. These participants were excluded from analysis. Two further participants were found to have very raised lymphocyte counts consistent with a diagnosis of chronic lymphocytic leukaemia and were excluded from further analyses. A further two participants had incomplete data leaving a final sample of 1027 participants. Table 1 shows the mean, median, minimum and maximum values by sex for each cell count. All distributions differed significantly from a normal distribution except red cell count in men. There was no significant difference between men and women for basophil counts. Women had significantly higher lymphocyte and platelet counts, though the difference, albeit statistically significant, was small for lymphocyte counts; for all other blood cells men had significantly higher counts (Table 1). Figure 1 shows the Q-Q plots against a normal distribution for erythrocyte, leukocyte and platelet counts by sex. The charts allow the lower and upper values where observed cell count data coincide with a theoretical normal distribution of cell counts to be determined. Table 2 displays the lower and upper limits for each blood cell count by sex. Deviation from a normal distribution is greater in the mid range for blood cells with lower counts such as eosinophils and basophils so that a 'normal' range is more arbitrary. The lower values for all cell types are very similar for both men and women except for red cell count. Inspecting the plots for red cell, total white cell and platelet counts, a clear outlier (not lying on the trajectory of the general curve/line) can be observed for female red cell counts. This outlying participant was excluded from further analyses.

**Table 1:** Blood cell counts by sex

		Red cells	Total white cells	Neutro-phils	Lympho-cytes	Mono-cytes	Eosino-phils	Baso-phils	Platelets
Men	Mean	4.8	7.1	4.6	1.8	0.57	0.18	0.04	257
	Median	4.8	6.9	4.3	1.7	0.54	0.14	0.04	254
	Min	3.3	2.5	1.4	0.5	0.18	0.0	0.01	107
	Max	5.9	14.1	10.9	4.4	1.73	1.15	0.34	507

Women	Mean	4.5	6.8	4.3	1.9	0.48	0.15	0.05	292
	Median	4.4	6.6	4.1	1.8	0.45	0.12	0.04	286
	Min	3.3	3.0	1.6	0.6	0.11	0.0	0.0	131
	Max	6.6	15.5	12.1	4.1	1.38	1.41	0.52	661

	p-value	< .001	.002	< .001	< .001	< .001	< .001	.12	< .001

Blood cell counts in 519 male and 508 female LBC1936 participants at mean age 69.5 years with p-value for difference (Mann-Whitney U test). Red cell count x10^12^/L, basophils x10^6^/L, all other cell counts x10^9^/L.

**Table 2:** Normal blood cell count ranges by sex

		Red cells	Total white cells	Neutro-phils	Lympho-cytes	Mono-cytes	Eosino-phils	Baso-phils	Platelets
Men	Min	4.2	5.0	2.8	1.2	0.40	0.10	0.03	210
	Max	5.5	11.1	7.5	2.6	0.80	0.38	0.09	370

Women	Min	3.7	5.0	2.8	1.1	0.35	0.10	0.03	210
	Max	5.4	9.6	6.0	2.9	0.65	0.40	0.11	380

Upper and lower blood cell counts values coinciding with a normal distribution in 519 male and 508 female LBC1936 participants at mean age 69.5 years. Red cell count x10^12^/L, basophils ×10^6^/L, all other cell counts ×10^9^/L.

### Relationships between blood cell counts: exploratory analyses

Table 3 shows Pearson correlation coefficients between the different blood cell counts by sex; Spearman rho values were very similar (data not reported here). More than half of the correlation coefficients reached significance, with significant correlation coefficients ranging from quite small at 0.11 to quite large at 0.50. Differences between men and women can be examined by comparing across the table diagonal. We did not formally test for significant differences between correlation coefficients in men and women at this stage because of the likelihood of Type 1 statistical errors. Rather, to elucidate relationships between different blood cell counts, principal components were extracted and rotated using a Varimax algorithm. Since the descriptive data indicated that men and women may have distinct processes regulating blood cell counts separate principal components analyses with Varimax rotation were therefore performed for men and women. Both analyses produced three principal components, accounting for a total of 59.5% of total variance in men and 65.5% of total variance in women. Table 4 shows the component matrices. The component structures appear different between men and women. Key differences, looking at values > 0.4, include a stronger positive relationship between neutrophil and lymphocyte counts in women than in men (Component 1), platelet count relates positively to eosinophil and basophil counts in women (Component 2) and men have a stronger positive relationship between red cell, neutrophils and monocyte counts (Component 3). We therefore sought to confirm whether these differences were due to a different structure of relationships between blood cell counts in men and women.

**Table 3:** Correlations between blood cell counts by sex

	Red cells	Neutrophils	Lymphocytes	Monocytes	Eosinophils	Basophils	Platelets
Red cells		**.19**	**-.01 NS**	**.02 NS**	**.02 NS**	**.06 NS**	**-.03 NS**
Neutrophils	.18		**.32**	**.50**	**.14**	**.19**	**.29**
Lymphocytes	.02 NS	.05 NS		**.59**	**-.03 NS**	**-.03 NS**	**-.02 NS**
Monocytes	.11	.39	.28		**.15**	**.15**	**.17**
Eosinophils	.05 NS	.08 NS	.28	.25		**.25**	**.21**
Basophils	.03 NS	.22	.16	.22	.25		**.26**
Platelets	-.02 NS	.28	.05 NS	.16	.13	.21	

Pearson correlation coefficients between blood cell counts in 519 male and 508 female LBC1936 participants. Correlation coefficients for men are in the bottom left section of the Table and those for women in the top right section in bold text. Non-significant (NS) p > .05 marked.

**Table 4:** Principal components of blood cell counts by sex

	Men			Women		
	1	2	3	1	2	3
Red cells	-.02	-.12	.86	.02	-.02	.97
Neutrophils	.05	.64	.53	.66	.34	.31
Lymphocytes	.77	-.15	.05	.85	-.19	-.08
Monocytes	.53	.32	.42	.87	.17	-.02
Eosinophils	.66	.15	-.04	.01	.66	-.02
Basophils	.46	.47	-.05	.03	.70	.12
Platelets	.01	.81	-.11	.12	.70	-.08

Rotated principal components of blood cell counts in 1027 LBC1936 participants separate analyses by sex.

### Relationships between blood cell counts: confirmatory analyses

For confirmatory analyses we hypothesised three latent variables corresponding to factor structure that most clearly separated cell counts which occurred in women. We performed structural equation modelling on all seven blood count variables, separately for men and women. We constructed the model so that Factor 1 related to neutrophils, lymphocytes and monocytes, Factor 2 related to eosinophils, basophils and platelets, Factor 3 related to red cells. In women the model fitted with marginal adequacy (RMSEA .074, 90% upper limit 0.80, Hoelter index 226), but fitted poorly in men (RMSEA .14, Hoelter index 68). We then explored the structure in more detail looking at each Component in turn. We excluded participants with anaemia by considering only those who had red cell counts within the normal distribution ranges (as shown in Table 2) in case this was influencing the clear separateness of red cell count. The remainder comprised 473 men and 500 women. First we considered Component 1, hypothesising a latent variable related to lymphocyte, monocyte, eosinophil and basophil counts as suggested for men in exploratory principal components analysis (Table 4). For men, the model had an adequate fit (Chi-square = 4.24, 2df, p = .12, RMSEA = .049, NFI = .967), whereas for women the model had a poor fit (Chi-square = 31.7, 2df, p < .001, RMSEA = .17, NFI = .893). Next we considered Component 2, hypothesising a latent variable related to neutrophil, basophil and platelet counts as suggested for men in exploratory principal components analysis (Table 4). For men, the model had a marginally adequate fit (Chi-square = 2.60, 1df, p = .11, RMSEA = .058, NFI = .967), whereas for women, the model had a poorer fit (Chi-square = 6.09, 1df, p = .014, RMSEA = .10, NFI = .927). Finally, we considered Component 3. A model with a latent variable related to red cell, neutrophil and monocyte counts, as suggested by Table 4, provided a poor fit for men (Chi-square = 76.9, 1df, p < .001, RMSEA = .11, NFI = .791), and a simpler model comprising red cell and neutrophil counts provided no better fit. Hence, Component 3 was confirmed as essentially identical to red cell count as suggested by the initial principal components analysis for women.

## Discussion

In this sample of over 1,000 participants aged about 70 years, significant differences between men and women in full blood count indices were detected. As expected, men had higher red cell counts than women, but they also had higher counts for other blood cell types except for basophils and platelets. For platelets, women had significantly higher counts than men. As Table 2 shows, these differences were not explained by participants whose cell count values fell beyond the normal range, rather, the normal ranges differed between sexes. Moreover, differences between men and women were not restricted to absolute count levels; the relative relationships between different cell types differed between sexes. For both men and women, three principal components emerged. One principal component identified in both men and women was defined by red cell count: this may be usefully considered as an 'erythropoietic' component. This component was also defined to a certain extent by neutrophil count. Another component might be considered as a 'leukopoietic' component which in men was defined by lymphocyte, monocyte, eosinophil and basophil counts, whilst in women, neutrophil, lymphocyte and monocyte counts loaded positively. The final component might be labelled a 'thrombopoietic' component. In men this was described by positive loadings of neutrophil, basophil and platelet counts, whilst in women it relates positively to eosinophil, basophils and platelet counts. Thus the key difference between men and women was found to be the relation between neutrophil counts and other blood cell counts. For men, neutrophil count is part of a 'thrombopoietic' trait whereas for women it is part of a 'leukopoietic' trait. It is important to note that although we have labelled these components as 'poietic', they may relate to processes of blood cell consumption and have nothing to do with bone marrow function or other aspects of cell production. There is a paucity of data on erythrocyte survival in older adults, but it does not appear to differ from young adults or between sexes [13].

Some limited data on the relationship between neutrophils and platelets are available from 34 healthy controls, mostly men, who participated in a study of the effects of type 2 diabetes on leukocyte and platelet counts [14]. Neutrophils, lymphocytes and monocyte counts were all lower in healthy controls than people with diabetes, this being more marked for neutrophils and monocytes than for lymphocytes. By contrast, platelet counts were higher in healthy controls than in people with diabetes. Although the direction of association is opposite in diabetes to that found in our sample, the presence of an association supports the hypothesis that there are underlying factors that drive changes in counts across a range of different blood cell types. Peripherally, one such factor that may link neutrophil and platelet populations is activation by injured atheromatous plaque [15]. Similarly, neutrophil-platelet interactions occur in septic shock [16], suggesting that inflammatory drivers may regulate changes across the haematological profile. There is a paucity of data that inform about these relationships in healthy older adults, but one study investigated nine centenarians, ten older adults (mean age 71 years) and ten younger adults (mean age 35 years) [17]. There were no significant differences between these small groups for erythroid burst-forming units or granulocyte-macrophagic colony-forming units, but centenarians had significantly lower ability to produce interleukin-3. These observations would be consistent with an independent erythropoietic factor. The study did not include data on either eosinophil or megakaryocyte colony forming cells, but alterations in interleukins would be expected to modulate relationships between these cells also if findings from disease states discussed above can be extrapolated to health. In addition to cytokine drivers, alterations in absolute and differential blood counts with ageing may relate to gene expression changes though, again, there is a paucity of data that inform about this [18]. Invoking possible mechanisms is highly speculative at present: further investigation is required into correlates of the different 'poietic' components in healthy adults, especially with regard to sex differences.

Our study has several limitations. First, it examined a narrow age cohort and thus is unable to shed light on possible changes with age. It is possible that haematological profiles and their underlying factor structure may differ in younger versus older populations: age-related changes in both haemoglobin concentration and platelet count are recognised [19,20]. Secondly, the sample was self-selecting as participation was voluntary. Moreover, participants had to be fit enough to travel to the clinical research facility for assessment. These factors introduce a natural sample bias towards more healthy participants; fortunately, we were interested in healthy participants to establish reference ranges rather than unhealthy participants. Thirdly, although our sample was 'healthy' in the conventional terms used for such studies [1,10,11], it included people with a wide range of stable medical conditions. Even though we excluded participants with clear haematological disorders, the mix of these conditions may have influenced the specific components that emerged. Fourthly, in addition to medical conditions, many participants were on regular medication which itself may have affected cell counts, although we were careful to exclude those on haematinics. Further data on healthy populations and at different ages are required to support the results of the present study.

## Conclusions

Two principal conclusions can be drawn from the data. First, normal ranges for haematological indices should be sex-specific; at present this only pertains to those associated with erythrocytes. The clinical relevance of different ranges may vary from disease to disease. Second, differences between individuals across a range of blood cell counts can be explained to a considerable extent by three major components, but these components are not the same in men and women. Changes in haematological profile are likely to reflect changes in one or more of these underlying factors.

## List of abbreviations

Hb: haemoglobin; IQ: intelligence quotient; LBC1936: Lothian Birth Cohort 1936; NFI: Normed Fit Index; Q-Q: quantile-quantile; RMSEA: Root Mean Square Error of Approximation

## Competing interests

The authors declare that they have no competing interests.

## Authors' contributions

JMS and IJD designed the study. AJG, CEB, JC and MT collected the data. JMS and RMcH analysed the data and drafted the manuscript. All authors contributed to and approved the final manuscript.

## Authors' information

RMcH is a Specialist Registrar in Geriatric Medicine in South East Scotland.

AJG is post-doctoral Research Fellow, University of Edinburgh Centre for Cognitive Ageing and Cognitive Epidemiology, Psychology.

CEB and JC are Research Associates, Department of Psychology, University of Edinburgh.

MT is Lecturer, Psychology, University of Edinburgh.

IJD is Professor of Differential Psychology and Director, University of Edinburgh Centre for Cognitive Ageing and Cognitive Epidemiology, Psychology.

JMS is Professor of Health & Ageing and Co-Director, University of Edinburgh Centre for Cognitive Ageing and Cognitive Epidemiology, Geriatric Medicine unit.

## Pre-publication history

The pre-publication history for this paper can be accessed here:

http://www.biomedcentral.com/1471-2326/10/4/prepub
